# Identification and analysis of key hypoxia- and immune-related genes in hypertrophic cardiomyopathy

**DOI:** 10.1186/s40659-023-00451-4

**Published:** 2023-08-09

**Authors:** Haozhen Yu, Lanxin Gu, Linfang Du, Zhao Dong, Zhuang Li, Mujun Yu, Yue Yin, Yishi Wang, Lu Yu, Heng Ma

**Affiliations:** 1 School of Basic Medical Sciences, Shaanxi University of Chinese Medicine, Xianyang, 712046 China; 2 University of Southern California, Los Angeles, CA 90089 USA; 3 Medical School of Yan’an University, Yan’an University, Yan’an, 716000 China; 4 Department of General Practice, Xijing Hospital, Fourth Military Medical University, Xi’an, 710032 China; 5 Department of Physiology and Pathophysiology, School of Basic Medicine, Fourth Military Medical University, Xi’an, 710032 China; 6 Department of Pathology, Xijing Hospital, Fourth Military Medical University, Xi’an, 710032 China

**Keywords:** Hypertrophic cardiomyopathy, Hypoxia, Immunity, Hub gene

## Abstract

**Background:**

Hypertrophic cardiomyopathy (HCM), an autosomal dominant genetic disease, is the main cause of sudden death in adolescents and athletes globally. Hypoxia and immune factors have been revealed to be related to the pathology of HCM. There is growing evidence of a role for hypoxia and inflammation as triggers and enhancers in the pathology in HCM. However, the role of hypoxia- and immune-related genes in HCM have not been reported.

**Methods:**

Firstly, we obtained four HCM-related datasets from the Gene Expression Omnibus (GEO) database for differential expression analysis. Immune cells significantly expressed in normal samples and HCM were then screened by a microenvironmental cell population counter (MCP-counter) algorithm. Next, hypoxia- and immune-related genes were screened by the LASSO + support vector machine recursive feature elimination (SVM-RFE) and weighted gene co-expression network analysis (WGCNA). Single-gene enrichment analysis and expression validation of key genes were then performed. Finally, we constructed a competing endogenous RNA (ceRNA) network of key genes.

**Results:**

In this study, 35 differentially expressed hypoxia genes were found. By using LASSO + SVM-RFE analysis, 10 more targets with differentially expressed hypoxia genes were identified. The MCP-count algorithm yielded five differentially expressed immune cells, and after assessing them for WGCNA characteristics, 612 immune genes were discovered. When hypoxia and immune genes were combined for cross-tabulation analysis, three hypoxia- and immune-related genes (*ATP2A2*, *DDAH1*, and *OMA1*) were identified.

**Conclusion:**

Based on hypoxia characteristic genes, three key genes were identified. These were also significantly related to immune activation, which proves a theoretical basis and reference value for studying the relationship between HCM and hypoxia and immunity.

**Graphical Abstract:**

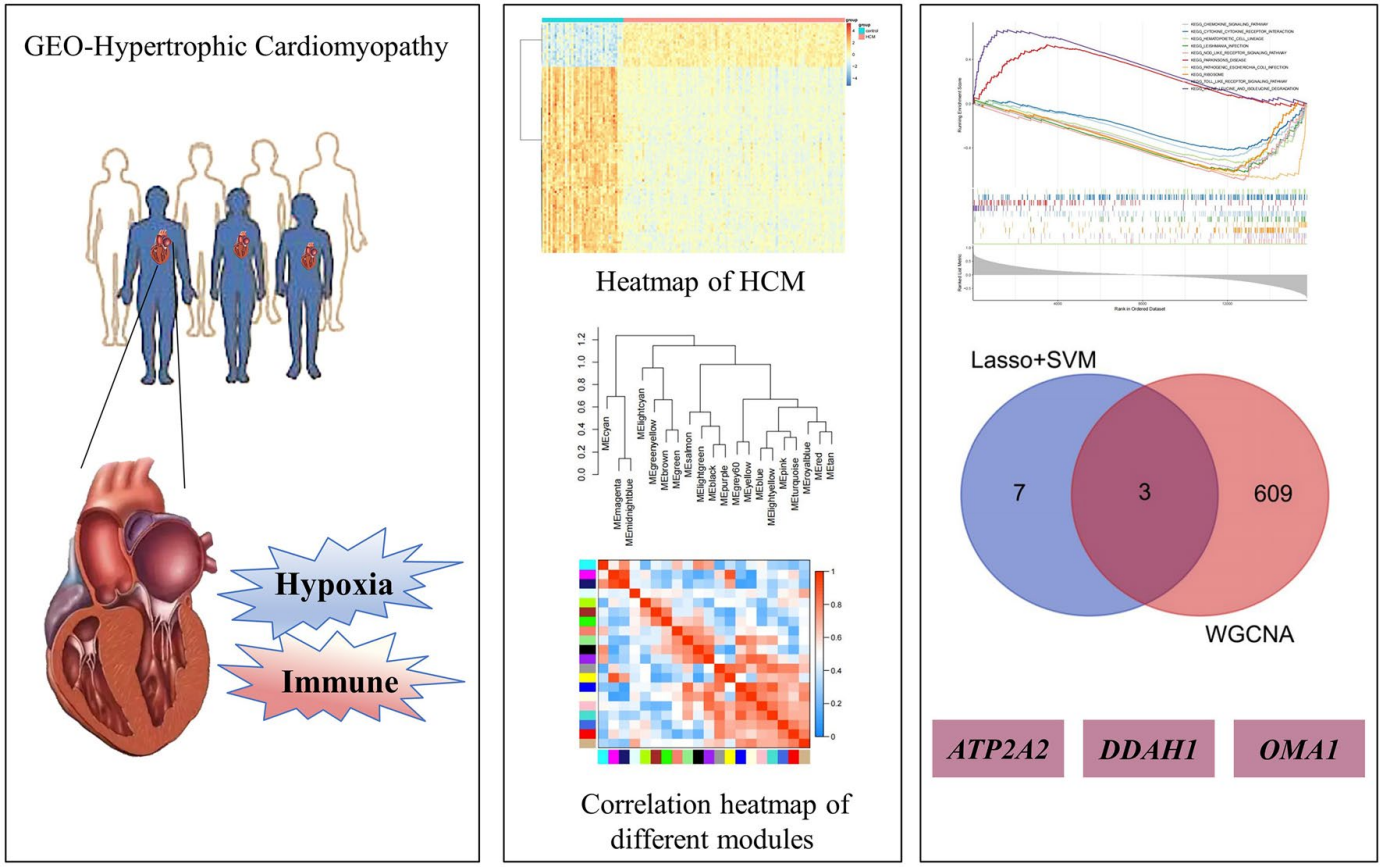

**Supplementary Information:**

The online version contains supplementary material available at 10.1186/s40659-023-00451-4.

## Background

Hypertrophic cardiomyopathy (HCM) is a primary disease involving myocardial fiber hypertrophy, disarrangement, and myocardial hypertrophy, especially asymmetric left ventricular hypertrophy. HCM is a prevalent inherited cardiovascular disease with diverse genotypes, phenotypes, clinical symptoms, and natural histories [1]. There are both genetic heterogeneity and non-genetic factors in hypertrophic cardiomyopathy [2]. The development of HCM depends not only on primary sarcomere damage caused by mutations but also on secondary disease-related alterations in the heart [3]. Epidemiological studies have estimated HCM prevalence as 0.2% in the general population. The continuous advancement in examination techniques has improved the diagnosis of cardiomyopathy, particularly through the broad application of echocardiography [4]. However, cardiomyopathy treatments remain inadequate, and no specific approaches have proven to improve prognosis. Moreover, few randomized studies on cardiomyopathy management have been published.

Hypoxia is a universal stimulus that impacts multiple biological processes and reduces oxygen availability (O_2_) [5]. Hypoxia is a defining physiological feature of several diseases, such as cancer, cardiovascular disease, and retinopathy [6]. It actively contributes to the pathological processes of these diseases by influencing the expression of various genes. Previous research has shown that hypoxia can control innate and adaptive immunity by regulating immune cell proliferation, development, and effector function [7]. Conversely, hypoxia can promote tissue dysfunction and disease development through immune cell dysregulation in pathological immune niches [8].

The immune system is involved in maintaining the normal physiological function of the heart and may play a crucial role in HCM. The immune system can drive aberrant inflammatory reactions and myocardial remodeling after injury [9, 10], and studies have shown that the myocardium of HCM patients exhibits inflammatory cell infiltration and fibrosis[11]. Cardiac hypertrophy is a pathogenic stimulus that impairs cardiac function by triggering inflammatory signal transduction and immune cell activation [11]. It is unclear whether this process is independent or somehow coordinated with primary pathogenic sarcomere gene mutations, thus interfering with the clinical HCM course and its prognosis. Therefore, we attempted to identify the critical pathogenic HCM pathways and pathophysiological mechanisms associated with non-myocyte mutations [12]. We employed a bioinformatics analysis to screen and identify hypoxia- and immune-related genes and relevant mechanisms for HCM diagnosis and treatment.

## Materials and methods

### Data source

The Gene Expression Omnibus (https://www.ncbi.nlm.nih.gov/geo/) database from the National Center for Biotechnology Information was searched for publicly available studies and samples that fulfilled the following criteria: (1) gene expression data series containing HCM heart tissue and normal heart tissue samples, (2) data from *Homo sapiens*, and (3) sample information that was consistent and without missing values. Four gene expression profiles (GSE36961, GSE141910, GSE143786, and GSE188324) were identified and collected for further analysis.

Firstly, GSE36961 (containing 106 HCM and 39 normal cardiac tissues samples) was used for differential expression analysis and WGCNA as a training set. The downloaded microarray data from GSE36961 was conducted with the fastlo normalization in advance, hence genes with the highest expression levels were retained to remove duplicate gene symbols for follow-up analysis. GSE141910 (containing 28 HCM and 166 normal cardiac tissues samples) and GSE130036 (containing 28 HCM and nine normal cardiac tissues samples) were selected as the validation sets for key gene expression to eliminate the influence of small sample size in control cohorts in the training set. Considering the raw gene counts in the high-throughput RNA-sequencing (RNA-seq) data of GSE141910 had been transformed using the LIMMA-Voom method, differential expression analysis of key genes was performed after removing duplicate gene symbols. Simultaneously, 56 HCM and 18 normal samples were randomly selected from GSE141910 and GSE130036 datasets after batch corrections for key gene verification as well. For the construction of a competing endogenous RNA (ceRNA) network targeting key genes, two plasma-related microarray datasets, that is, GSE188324 (containing the 12 HCM and 11 normal plasma samples) and GSE143786 (containing 14 HCM and seven normal plasma samples) were employed. After processing the miRNAs-related data in GSE188324 using a log-ratio transformation, differentially expressed analysis was conducted to screen differentially expressed miRNAs. LncRNAs expression profiles in GSE188324 were downloaded to obtain differentially expressed lncRNAs.

Hypoxia-associated genes from the human disease spectrum were downloaded from the UniProt database (https://www.uniprot.org/help/uniprotkb); the data were de-duplicated to ultimately obtain 493 hypoxia-associated genes (shown in Additional file 1).

### Identification of differentially expressed hypoxia-related genes

The “limma” R package (v3.44.3) [13] was used to identify differentially expressed genes (DEGs) between HCM and normal tissues in the GSE36961 dataset with a cutoff value of |log2fold change| > 0.5 and a false discovery rate (FDR) < 0.05, which were visualized using the “ggplot” (v3.3.2) [14]and “heatmap” R packages (v0.7.7). These were further intersected with the 493 hypoxia-related genes to obtain differentially expressed hypoxia-related genes. The results were used to create a Venn diagram.

### Screening target genes by machine learning analysis

In order to obtain the potential important genes for diagnostic purposes in the GSE36961 dataset, the Least Absolute Shrinkage and Selector Operation (LASSO) and Support Vector Machine-Recursive Feature Elimination (SVM-RFE) algorithms were employed [15], and the overlapping genes obtained from the two algorithms were used as target genes.

### The MCP-counter algorithm inferred the immune cell abundance

To investigate the relationship between immunity and disease and thus screen for immune genes associated with HCM, the absolute abundance of eight immune cell types, one fibroblast, and one epithelial cell in 106 HCM samples was quantified based on the normalized transcriptome data using the MCP-counter algorithm [16]. The expression profiles of immune cell-specific marker genes in each sample were converted to the abundance scores, which were not directly interpreted as cell fractions, but displayed the high correlations between the estimated score and the real cell fractions. The expression levels of various cells listed above in 106 samples were visualized as heatmaps, and the Wilcoxon test was used to calculate the expression difference for each cell type between HCM and normal tissues (*P* < 0.05).

### Identification of immune-related genes based on weighted gene co-expression network analysis (WGCNA)

WGCNA [17] analysis was used to locate co-expressed gene modules, investigate the relationships between modules and traits or phenotypes, and choose the network’s core genes and highly associated gene modules. First, we determined whether the samples of genes required filtering by using the “goodSamplesGenes” function in the “WGCNA” package (v1.69). An adjacency matrix was then built using Pearson’s correlation analysis for all gene pairings, and adjacency matrix was utilized to build a scale-free co-expression network based on a soft-threshold value, which favors strong gene–gene correlations and penalizes weak correlations. The adjacency matrix was then converted into a topological overlap matrix (TOM) [18], which compares the weighted correlation between two nodes and other nodes to represent the similarity of nodes quantitatively. Then, using hierarchical clustering (minModuleSize = 30), we determined that each module must include at least 30 genes. Finally, we determined the feature genes, carried out hierarchical module clustering, and combined related modules (abline = 0.25).

In this study, we used two different techniques to pinpoint key clinical feature-related modules. The expression pattern of the module in each sample was described by the module signature genes (ME), which stand in for the module’s first main component. Additionally, the value of each gene (GS = lgP) in a linear regression of mediated gene expression versus clinical characteristics was identified as the gene significance (GS). The average GS of all the genes in the module was then used to define module significance (MS). The ability of MS to integrate clinical data into the co-expression network was assessed. MS was defined as the mean absolute GS calculated for all genes in a given module. Next, a Venn diagram was constructed to show the intersection of differentially expressed hypoxia genes and immune (key module) genes as the HCM hypoxia- and immunity-related genes.

### GSEA

In order to further explore the biological pathways related to the hypoxia- and immunity-related genes, the R software clusterProfiler package (version 3.16.1) [19] was used for GSEA. The correlations between each key gene and all other genes in training sets were first calculated, and all sorted genes were ranked based on the correlation coefficients from high to low and used as the tested geneset. Meanwhile, the “C5: GO: Gene Ontology gene sets” and “C2: KEGG subset” were used as reference gene sets to detect the enrichment of tGO and KEGG terms in the tested geneset. The threshold was set to |NES| > 1, p.adjust < 0.05, q < 0.25.

### Validation of the expression levels of hypoxia- and immune-related genes

To further investigate the expression levels of hypoxia- and immune-related genes in HCM, we validated the expression of key genes in GSE141910 and GSE130036 datasets, where 56 HCM and 18 normal myocardial tissues samples in two high-throughput RNA-seq datasets were randomly selected as a new combined dataset after batch corrections. Principle component analysis (PCA) was used to examine the distribution between different groups. The expression of the hypoxia- and immunity-related genes in GSE141910 and combined cohorts were then analyzed using the Wilcoxon test. *P* < 0.05 was considered statistically significant.

### Construction of ceRNA network based on key hypoxia- and immune-related genes

For the regulatory network targeting key hypoxia- and immune-related genes, the GSE188324 and GSE143786 datasets were used to obtain differentially expressed miRNAs and lncRNAs (|log2fold change| > 0.5 and *P* value < 0.05). Next, we used the miRWalk website (http://mirwalk.umm.uni-heidelberg.de/) to predict miRNAs involved with hypoxia- and immune-related genes. We then overlapped these predicted miRNAs with differentially expressed miRNAs, where miRNAs showing inverse expression trends to the hypoxia- and immune-related genes were screened. In the same way, the starbase website (http://starbase.sysu.edu.cn/) was used to predict the lncRNAs of the miRNAs in the same relationship pairs, and those that had opposite expression trends to key miRNAs were retained. Finally, the ceRNA network was visualized using the “Cytoscape” package (v3.7.2).

### Statistical analysis

All calculations and statistical analyses were performed using R software (v4.1.0). Differences in immune infiltration analysis and gene expression between different groups from online datasets were compared using Wilcoxon test. Quantitative results were examined using Prism 9.0 and represented as mean ± SEM. Transverse aortic constriction (TAC) model establishment and qRT-PCR analysis quantitative results were examined using Prism 9.0 and expressed as mean ± SEM. A two-sided *P* < 0.05 was considered statistically significant.

### Animal model

All applicable institutional and national guidelines for the care and use of animals were followed. All the animal experiments were approved by the Institutional Animal Care and Use Committee of the Fourth Military Medical University (IACUC-20,200,602). Mice were obtained from the Laboratory Animal Center of the Fourth Military Medical University. All animal experiments used 6–8-week-old male C57BL/6 mice. All experimental protocols were approved by the Animal Ethical Committee of the Fourth Military Medical University. The mice were kept in a temperature-controlled environment (22 ± 2 °C) at a 12-hour light/dark cycle. Food and water were provided *ad libitum*.

### Construction of the hypertrophic cardiomyopathy model

18-20 g male C57 mice were anesthetized with 50 mg/Kg body weight pentobarbital anesthetic. The mice were placed in a supine position and fixed on a heating pad to maintain body temperature. The neck and chest were depilated with hair removal cream and then sterilized. After endotracheal intubation, a ventilation tube was inserted and connected by a catheter to a small animal ventilator (HX-101E, Techman Soft Co., Chengdu, China) through the y-connector. The ventilation volume for mice was 2.4 mL, and the respiratory rate was maintained at 120 times/minute. When the respiratory rate of the animal had synchronized to the ventilator, the skin, pectoral muscle and intercostal muscle are cut from the second intercostal space of the heart, the chest expander was used to open the intercostal space, micro tweezers were used to separate the thymus and expose the aortic arch, a small section of 7.0 suture was then placed under the arterial arch between the brachiocephalic trunk and the left common carotid artery and a knot was tied around the arterial arch for standby, and a 27G flat head needle was then inserted into the knot and placed parallel to the artery. The live knot was tightly tied to the needle and the artery, then another knot was made on top of the first. After quickly taking out the needle, surgical stenosis of the distal aortic arch of the innominate artery with a theoretical diameter of 0.4 mm was obtained. After the thymus was reset and the intercostal muscles and skin sutured with 6.0 polypropylene suture in turn, the condition of the animal was analyzed and considered. If the animal was stable, the tracheal intubation tube was pulled out, and adhesive tape was remove from the fixed tooth tendons and limbs. Warm 0.2ml penicillin was injected intraperitoneally. If dehydration occurred, normal saline was injected intraperitoneally, and the animal put it back into its cage after waking up. Four weeks after the procedure, the animals were harvested, heart weight, body weight, and tibia length were recorded, and the relative heart weight, including heart weight/body weight (HW/BW) and heart weight/tibia length (HW/TL) was calculated (Additional file 2). LVS and LVEF were recorded by echocardiography (Additional file 9). The mouse heart group with a statistically significant HW/TL ratio and an echocardiographic LVEF of less than 50% suggested modeling results and could be verified by subsequent experiments.

### Validation of the expression levels of critical genes by RT-qPCR

cDNA from the left ventricle tissue harvested at the same time animals were sacrificed (sham and HCM, n = 6 per group) was extracted for qPCR. Primers spanning exon-exon junctions were designed in-house using Primer-BLAST (https://www.ncbi.nlm.nih.gov/tools/primer-blast/). *Gapdh* was used as a reference housekeeping gene. The qPCR assays were performed using FastStart Universal SYBR Green Master mix (Takara Biomedical Technology (Beijing) Co.) according to the manufacturer’s protocol with minor modifications. A 25-µL reaction (10 ng cDNA) was run in a 96-well plate (Axygen Scientific Inc., Silicon Valley, CA, USA) on a CFX96TMReal-Time System (BIO-RAD). The primer sequences are shown in Table 1.

**Table 1:** Primer sequences

Primer name	Sequence (5′-3′)
*atp2a2* (Mus)-F	TCACACCGCTGAATCTGACC
*atp2a2* (Mus)-R	ACTCCAGTATTGCGGGTTGT
*ddah1* (Mus)-F	TGCGTGTTCGTGGAGGACG
*ddah1* (Mus)-R	CCTCTTTCATCATGTCAACCTCCTT
*oma1* (Mus)-F	TCTCTGGAGTGAATAACCTGGC
*oma1* (Mus)-R *gapdh* (Mus)-F *gapdh* (Mus)-R	GCACTTGAGAGGCATCTTGATT GGTGAAGGTCGGIGTGAACG CTCGCTCCTGGAAGATGGTG

## Results

### Screening for differentially expressed hypoxia-associated genes

**Fig. 1:**
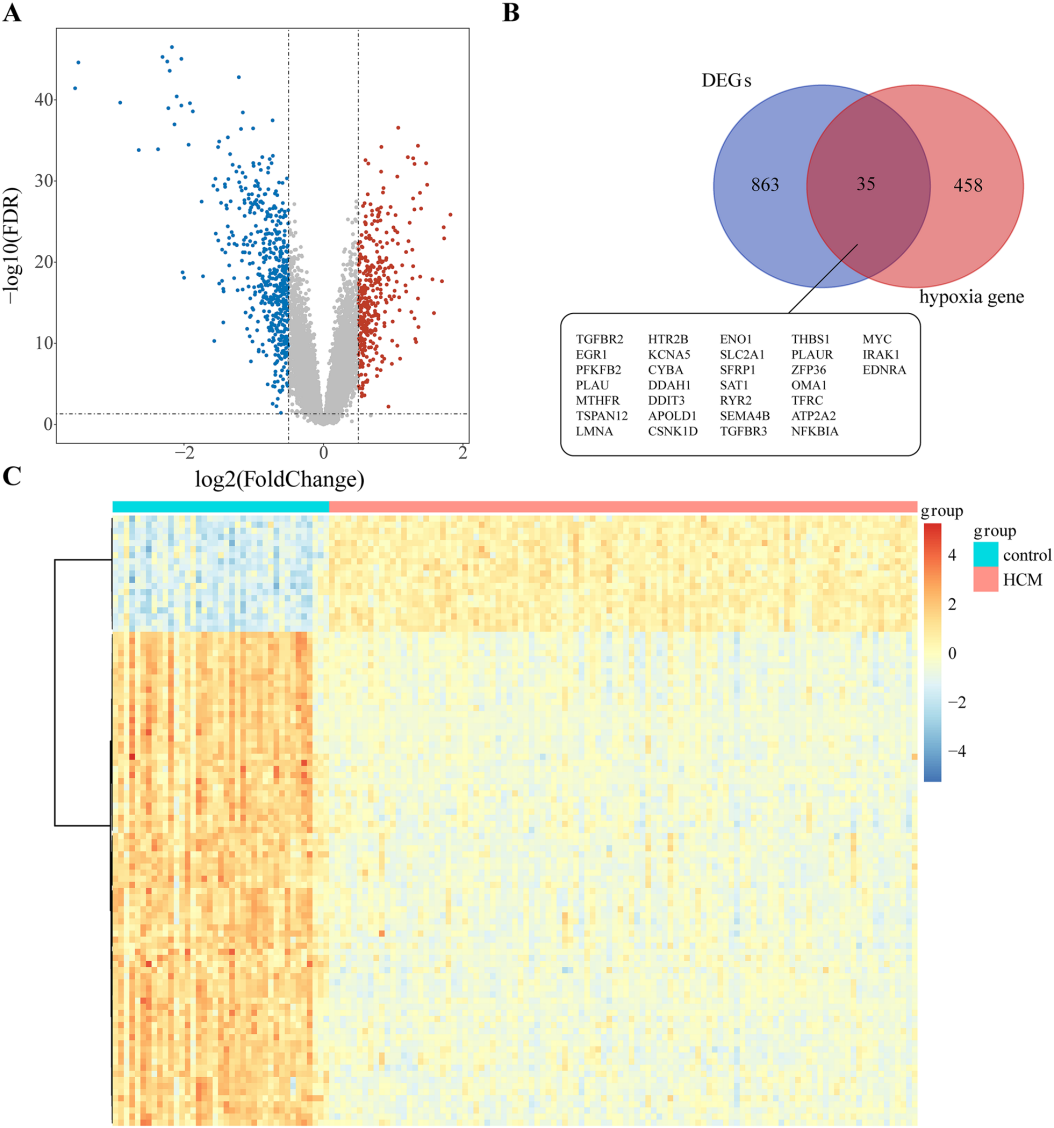
Identification of differentially expressed genes (DEGs) in HCM and normal groups. **A** Volcano plot of DEGs in samples from HCM and controls in GSE36961. Red dots represent up-regulated genes and blue dots represent down-regulated genes in HCM samples, gray indicates no significance. **B** Heatmap for DEGs in HCM (red) and controls (green). **C** Venn diagram of thirty-five hypoxia-related DEGs in HCM

The expression of 898 genes was determined to be significantly different between normal and HCM in the GSE36961 dataset. Of these, 372 genes were upregulated, and 526 genes were downregulated in HCM. The volcano map and heatmap are shown in Fig. 1A and B. Thirty-five hypoxia-related genes differentially expressed in HCM were identified by overlapping the 493 hypoxia genes determined above and the DEGs (shown in Fig. 1C).

### Screening target genes by LASSO regression and SVM-RFE analysis

**Fig. 2:**
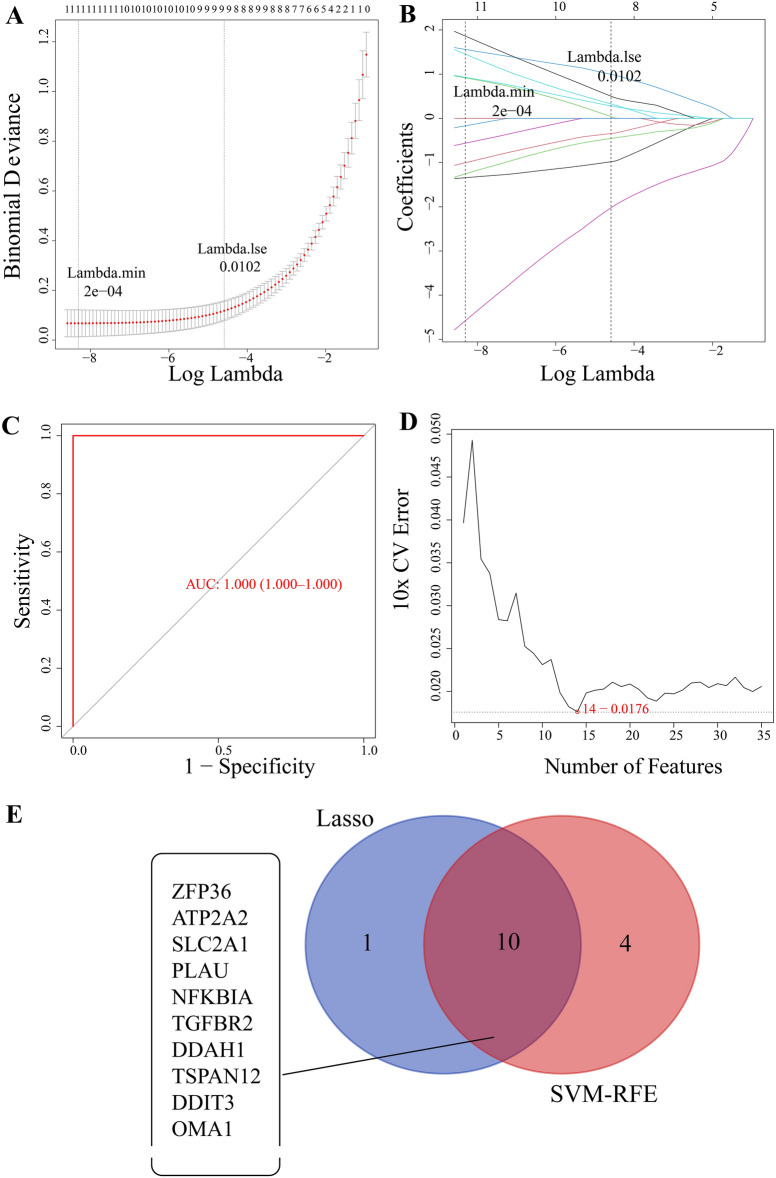
Targeted genes screening through LASSO regression and SVM-RFE analyses. **A** Error plot for the logistic LASSO regression coefficient. **B** 11 genes were screened in the logistic LASSO model. **C** ROC curve under the LASSO model. **D** Plot of generalization error versus the number of features. **E** Venn diagram of ten intersection genes

LASSO regression analysis, with the parameter family set to binomial, produced gene coefficient plots and error plots for cross-validation **(**Fig. 2A and B). The model was optimized to when the λ value was smallest, and 11 genes were identified out the 35 hypoxia-related genes found above (*Zfp36, Atp2a2, Slc2a1, Plau, Nfkbia, Tgfbr2, Ddah1, Ednra, Tspan12, Ddit3*, and *Oma1*). Each of the 11 genes has a unique coefficient **(**Additional file 3). Next, we generated a receiver operating characteristic (ROC) curve to validate the model’s functionality **(**Fig. 2C**).** HCM and normal samples could be identified with an AUC value of 1.000, showing that the model performed well. We also analyzed 35 differentially expressed hypoxia genes with SVM-RFE. We plotted the generalization error versus the number of features and used 10-fold cross-validation to select eigengenes (Fig. 2D). When the “sweet spot” was 0.0176, 14 characteristic genes were detected (*Zfp36, Atp2a2, Slc2a1, Plau, Nfkbia, Tgfbr2, Ddah1, Ednra, Tspan12, Ddit3, Oma1, Lmna, Penk, Myc, and Eno1*). Intersection of the genes identified by LASSO analysis and SVM-RFE analysis yielded 10 genes (*Zfp36, Atp2a2, Slc2a1, Plau, Nfkbia, Tgfbr2, Ddah1, Ednra, Tspan12, Ddit3*, and *Oma1*) as shown in the Venn diagram (Fig. 2E). From this figure, we can compare the similarities and differences between the two machine learning algorithms, LASSO and SVM, increasing the reliability of the diagnostic marker screening approach here.

### MCP-counting algorithm identified significantly different immune cells

**Fig. 3:**
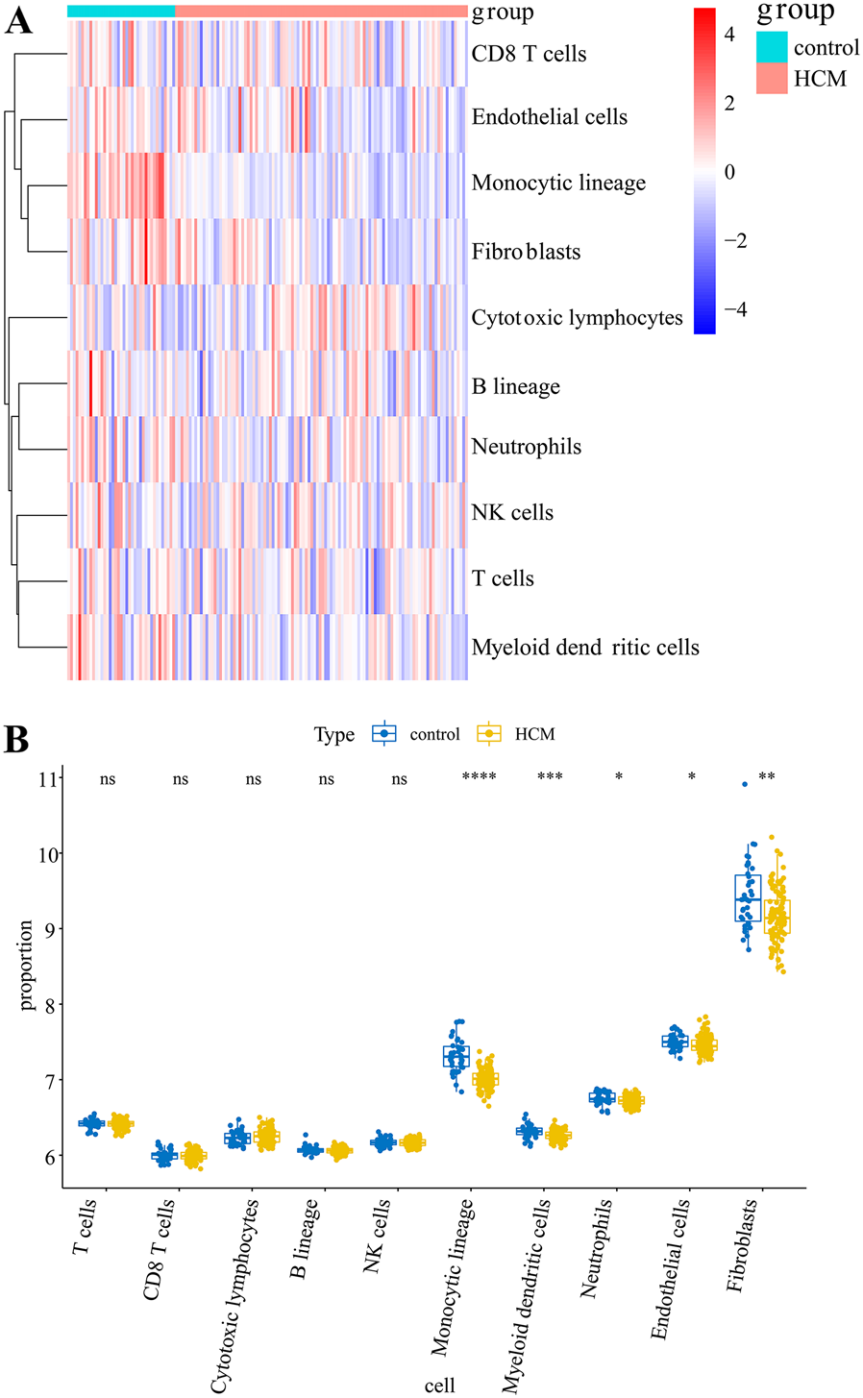
Immune cell abundance by the MCP-counting algorithm. **A** Heatmap of different immune cell contents through the MCP-counting algorithm. **B** Boxplots of cell contents across different groups under the MCP-counting algorithm (*P < 0.05, **P < 0.01, HCM vs. Control)

To study the relationship between disease and immunity, we used the MCP-counter algorithm to screen for the predominant cells types associated with normal and HDM samples in the in the GSE36961dataset. The genetic contents of 8 immune cells, fibroblasts and one type of epithelial cells are visualized as heatmaps (Fig. 3A) and boxplots (Fig. 3B). We found a substantial difference between normal and pathological tissue in the composition of five cell types (monocytic lineage, myeloid dendritic cells, neutrophils, endothelial cells, and fibroblasts), suggesting an important regulatory role for these cells in immune infiltration.

### Weighted co-expression network construction and key module identification

**Fig. 4:**
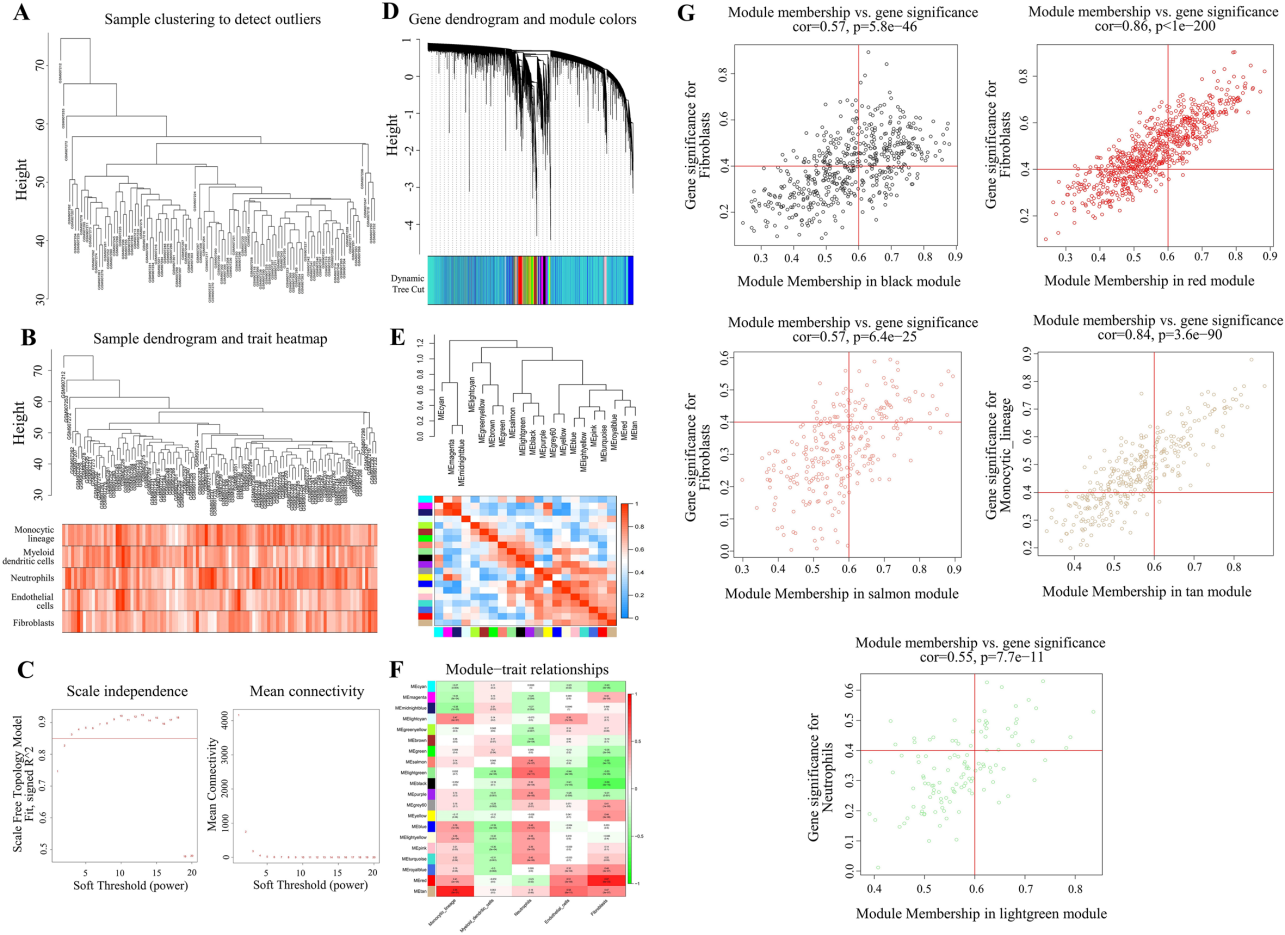
Weighted co-expression network construction analysis (WGCNA) based on five keyimmune cells. **A** Sample clustering dendrogram in GSE36961. **B** Merging data sample clustering and phenotypic information. **C** Analysis of the scale-free fit index (left) and the mean connectivity (right) for various soft-thresholding powers. **D** Module clustering dendrogram of all DEGs clustered based on a dissimilarity measure. **E** Cluster dendrogram and correlation heatmap of different modules. **F** Correlation heatmap of modules related to clinical features (Each cell contains the correlation coefficient and *P* value). **G** Critical module membership (MM) and gene significance (GS) scatter plot (vertical line for |MM|=0.6 and horizontal line for |GS|=0.4)

The samples in GSE36961 were grouped using the Pearson correlation coefficient. The five cell types found to be significant above were utilized as features to analyze the immune-related genes in HCM. As shown in Fig. 4A, we created a sample clustering tree and the corresponding heatmap of clinical characteristics (Fig. 4A, B), and no outliers needed to be removed. The ideal soft threshold was 3 (R2 = 0.85) from the scale-free soft-threshold distribution map (Fig. 4C). After dynamic tree trimming and average hierarchical clustering, 20 modules were ultimately identified, with gray modules denoting genes that did not fit into any of these modules (Fig. 4D). To identify modules related to the clinical features of HCM, we clustered a dendrogram of all DEGs (Fig. 4E) based on differential measures (1-TOM), and the heatmaps reflected the correlation between different modules and size distribution (Fig. 4F). For determining key module genes, we selected key modules under the following conditions: non-gray modules, P < 0.05 and correlation with any immune cell | cor | > 0.5. As shown in Fig. 4G, five modules were screened: salmon, light green, black, red, and tan. We then screened for module membership (MM) and GS, generated a scatter plot, and screened out key module genes based on the criteria of |GS| > 0.4 and |MM| > 0.6 (Fig. 4G). As a result, 612 key module genes were identified that represent the most closely related to immune function in HCM (Additional file 4).

### GSEA of hypoxia- and immune-related genes

**Fig. 5:**
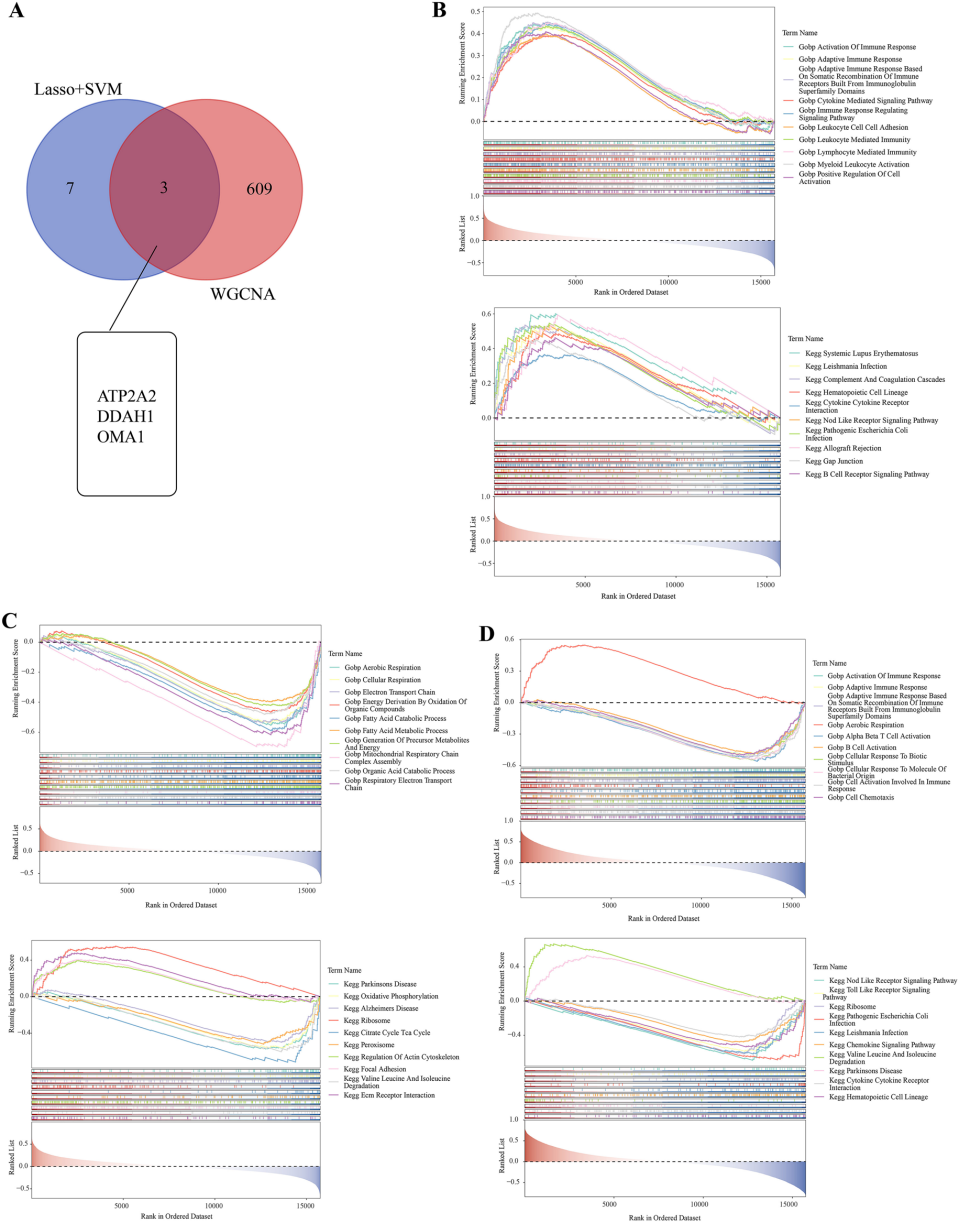
Analysis of hypoxia- and immune-related genes using gene set enrichment analysis (GSEA). **A** Venn diagram of hypoxia- and immune-related genes. **B** GO/KEGG enrichment analyses using GSEA for the *ATP2A2* gene (top10). **C** GO/KEGG enrichment analyses using GSEA for the *DDAH1* gene (top10). **D** GO/KEGG enrichment analyses using GSEA for the *OMA1* gene (top10)

Using a Venn diagram, the intersection of the hypoxia-related genes screened by LASSO + SVM-REF and the immune genes screened by WGCNA produced three hypoxia-immunity-related genes (Fig. 5A). The GO and KEGG pathways for these three hypoxia-immunity-related genes (*ATP2a2, DDAH1*, and *OMA1*) were identified using GSEA (Fig. 5B–D). We provide the top 10 entries for each gene in GO and KEGG individually. For example, *Atp2a2* was enriched in 717 related GO entries and 45 KEGGs, while *Ddah1* was enriched in 569 related GO entries and 30 KEGGs. In total, 1296 related GO entries and 72 KEGGs were enriched for *Oma12*.

Interestingly, *ATP2A2* and *DDAH1* share three GO entries: activation of immune responses, adaptive immune responses, and responses based on spontaneous recombination of immune receptors with immunoglobulin superfamily structures, though the former is mainly enriched in leukocyte-related entries, while the latter is enriched in cell-related entries. The *OMA1* entry is mainly fat metabolism-related. However, the three genes with KEGG entries are very different (Additional file 5).

### Validation of the expression levels of the hypoxia- and immune-related genes

**Fig. 6:**
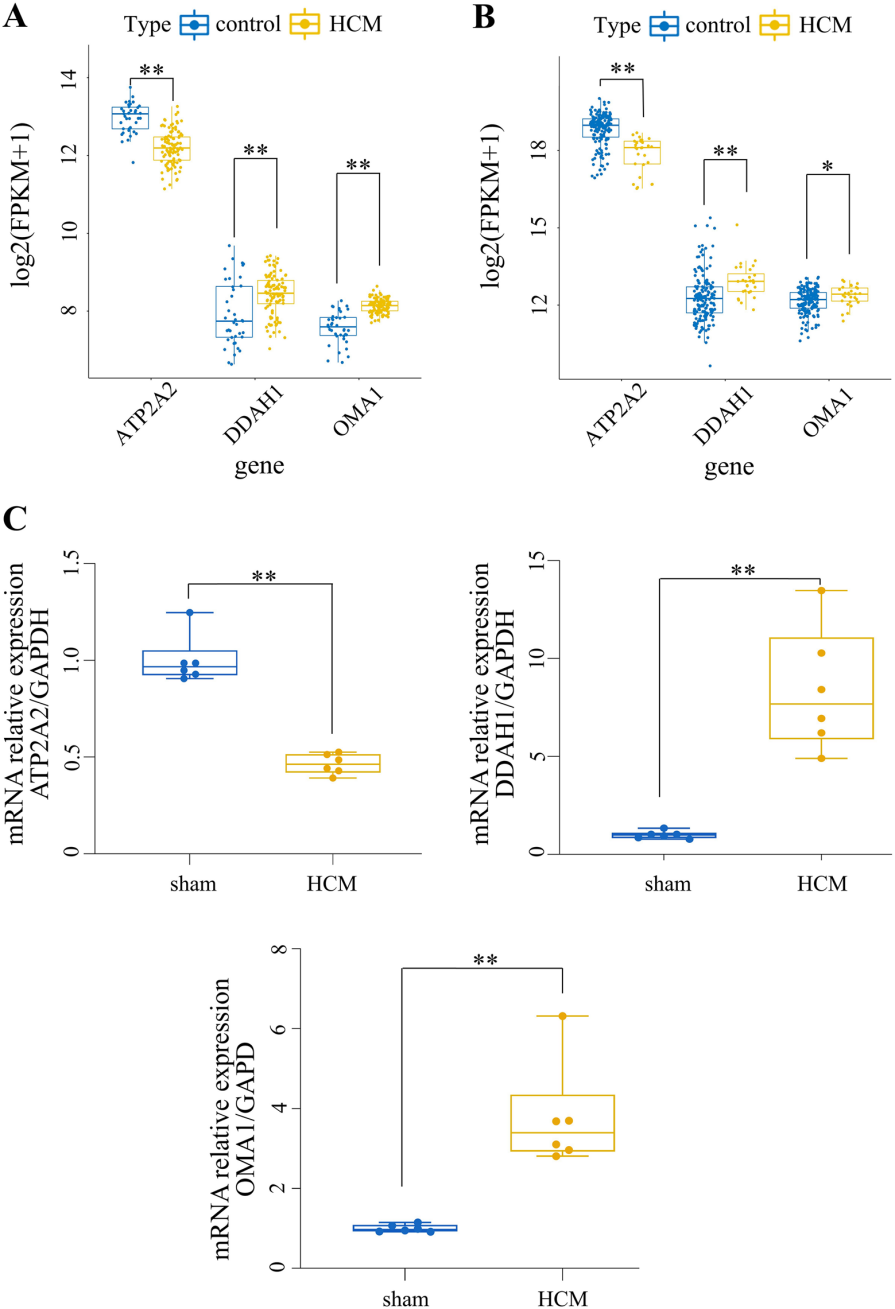
Validation of hypoxia and immune-related gene expression. **A** GSE36961 boxplot. **B** GSE141910 boxplot (*P < 0.05, **P < 0.01, HCM vs. Control). **C** qRT-PCR verification for critical genes. Values represent means ± SEM, n = 6/group, **P < 0.01 vs. Sham group

To validate the expression of hypoxia- and immune-related genes in HCM, the GSE36961, GSE141910 and GSE130036 datasets were used. The results of GSE141910 and GSE130036 before and after batch correction are shown in Additional file 6A, B, documenting diverse distributions of samples from different sources. The expression of three hypoxia- and immune-related genes in GSE36961, GSE141910, and combined cohorts are displayed in Fig. 6A, B and Additional file 6C, revealing that the expression trends of *ATP2A2* and *DDAH1* were consistent across the three datasets, in which *ATP2A2* expression was higher in the normal group than in HCM, *DDAH1* expressed higher in HCM than in the normal group. *OMA1* expression had distinct differences between two groups in GSE36961 and GSE141910, while there was no significantly alterations in the combined cohorts. Similarly, the area under the curve (AUC) value of the ROC curve for *ATP2A2* (0.89) and *DDAH1* (0.902) rather than *OMA1* (0.546) suggested excellent prognostic accuracy for HCM (Additional file 6D).

Using the animal model established above, the mouse heart group with statistically significant HW/TL ratio (Additional file 2) and echocardiographic LVEF below 50% (Additional file 9) was selected as the HCM group, and the left ventricular tissue samples from the normal and HCM groups were verified by qRT-PCR. Again, the expression trends of the three genes were consistent between the mouse model and the dataset validation results (Fig. 6C).

### Construction of ceRNA network based on three hypoxia- and immune-related genes

**Fig. 7:**
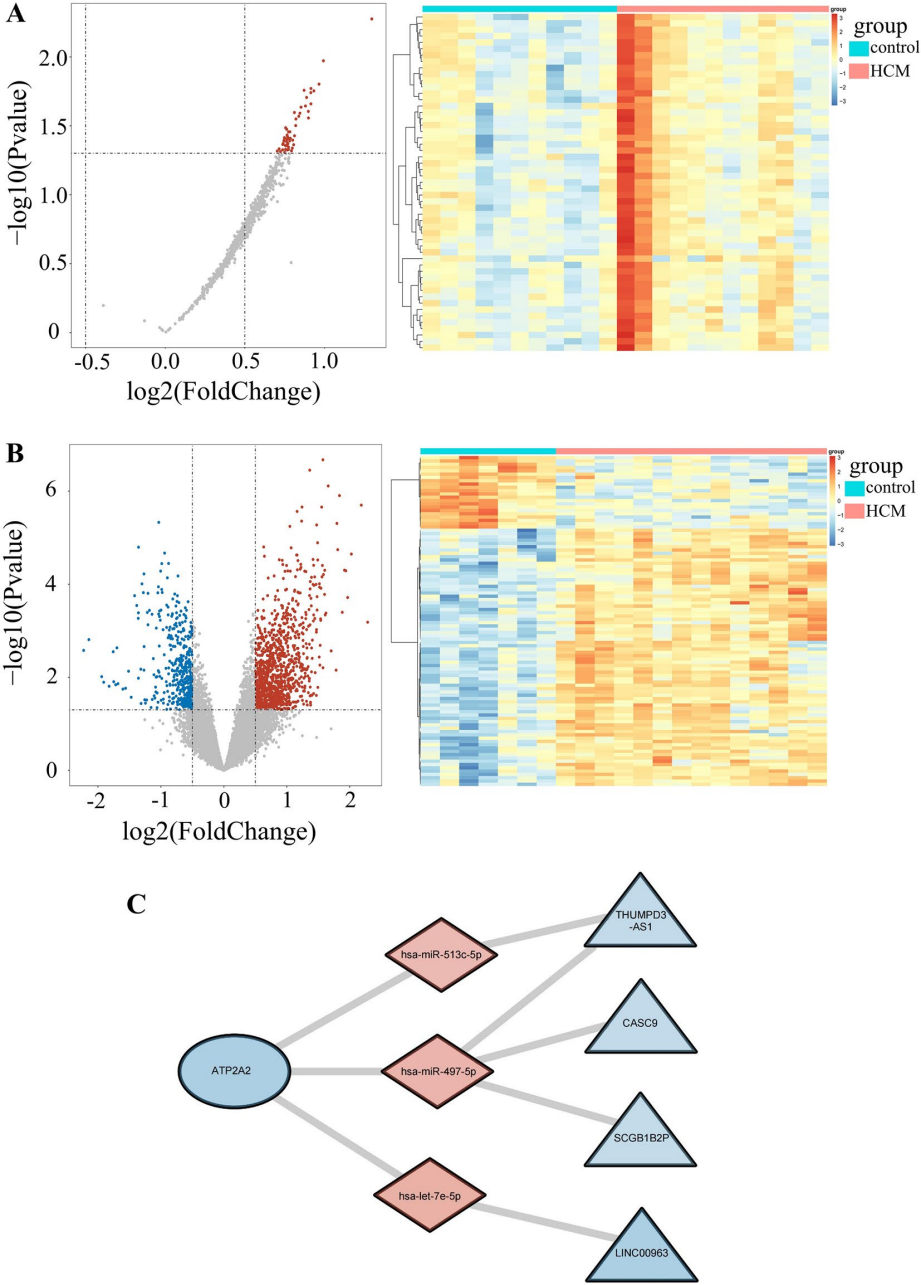
ceRNA network construction. **A** Heatmap and volcano plot of differentially expressed miRNAs (top100) between HCM and control samples in GSE188324. **B** Heatmap and volcano plot of differentially expressed lncRNAs (top100) between HCM and control samples in GSE143786. For volcano plot, red dots represent up-regulated genes and blue dots represent down-regulated genes in HCM samples, gray indicates no significance. **C** The mRNA-miRNA-lncRNA network diagram. Blue oval indicates the key hypoxia and immune-related gene, pink diamond indicates miRNA, blue trangle indicates lncRNA

Based on the GSE188324 and GSE143786 microarray data in the GEO database, we identified 53 differentially expressed miRNAs (all upregulated) (Fig. 7A) and 1,561 differentially expressed lncRNAs (Fig. 7B) (1115 upregulated and 446 downregulated). These differentially expressed genes were used to construct a ceRNA network.

Through the miRWalk website, we also predicted the miRNAs from the three hypoxia- and immunity-related genes, resulting in 2224 predicted miRNAs (Additional file 7). In addition, we selected hub gene-miRNA relationship pairs with opposite expression trends; there were four matching miRNAs, namely, *hsa-miR-513c-5p*, *hsa-let-7e-5p*, *hsa-miR-371a-5p*, and *hsa-miR-497-5p*. Similarly, we obtained 643 predicted lncRNAs using the starbase website to predict the lncRNAs of the miRNAs that matched the previous step (Additional file 8). Again, the miRNA-lncRNA relationship pairs with opposite expression trends were selected; there were three matching lncRNAs, namely *THUMPD3-AS1*, *CASC9*, *SCGB1B2P*, and *LINC00963*. Finally, the network included eight nodes (one mRNA, four lncRNAs, and three miRNAs screened, as shown in the appendix) and eight edges. The network map of ceRNA was visualized with Cytoscape software (v3.7.2; Fig. 7C). The map revealed that *ATP2A2* plays an important role in HCM and could be used as a diagnostic marker for the disease.

## Discussion

We first downloaded the GSE36961 dataset and performed the differential analysis with the filtering criterion FDR < 0.05 and |log2FC| > 0.5 to acquire 898 DEGs. Thirty-five hypoxia-related DEGs were identified by intersecting 493 hypoxia-related genes and 898 DEGs. The gene numbers were further condensed using LASSO and SVM analyses of the GSE36961 dataset, ultimately identifying 11 and 14 genes, respectively. Ten common genes were obtained by taking the intersection of the two analyses (*ZFP36*, *ATP2A2*, *SLC2A1*, *PLAU*, *NFKBIA*, *TGFBR2*, *DDAH1*, *TSPAN12*, *DDIT3*, and *OMA1*). We also focused on the relationship between disease and immunity. Using the MCP-counting algorithm, we found that the composition of normal and HCM samples varied considerably in five cell types. WGCNA was performed using these cells to identify five immune-related modules (non-gray modules with P < 0.05 and the correlation |cor| > 0.5 with any one immune cell). A total of 612 critical module genes were screened with the criterion of |GS| > 0.4 and |MM| > 0.6. Finally, the common and critical module genes were intersected to obtain three hypoxia- and immune-related genes: *ATP2A2*, *DDAH1*, and *OMA1*.

ATPase sarcoplasmic/endoplasmic reticulum Ca^2+^ transporting 2 (*ATP2A2* [Ca^2+^-ATPase 2]) helps maintain low cytoplasmic Ca^2+^ concentrations. Decreased contractility of hypertrophied cardiomyocytes significantly contributes to ventricular dysfunction in failing hearts. A crucial feature of the failing heart is the reduced *ATP2A2* content and activity, which might explain some physiological flaws in hypertrophied cardiomyocytes. The flaws play a significant role in progressing from compensatory hypertrophy to heart failure [20]. In contrast, *DDAH1* is crucial in reducing myocardial hypertrophy and ventricular remodeling under stress conditions [21]. Furthermore, it is critical in attenuating left ventricular remodeling after AMI by regulating intracellular ROS levels and apoptotic sensitivity through a SOD2-dependent pathway [22]. *DDAH1* up-regulation might represent a promising therapeutic strategy against angiotensin II-induced end-organ damage [23]. Finally, OMA1 zinc metallopeptidase (*OMA1)* is the essential regulator of mitochondrial morphology and cardiomyocyte survival. Heart failure, cell death, and mitochondrial fragmentation can be rescued by knocking down *Oma1* in mice [24]. *OMA1* is an essential mediator of multiple etiologies of heart failure and a possible therapeutic target for maintaining myocardial integrity [25]. While these genes can regulate cardiac function, their cardioprotective effects were not explored from the perspective of hypoxia and immunity.

We performed a single-gene GSEA enrichment analysis in this study based on the hypoxia- and immune-related genes we identified. In addition, we examined the GSE141910 gene expression profiles as a validation set. Finally, a ceRNA network for hypoxia- and immune-related genes was constructed based on the GSE188324 and GSE143786 datasets. Prior research has shown that *ATP2A2* is associated with cancer risk. Higher *ATP2A2* expression is correlated with a better prognosis in patients with astrocytoma, as *ATP2A2* can suppress tumor growth [26]. *ATP2A2* can upregulate the expression of *Hsa-miR-497-5p* miRNA and downregulate *CASC9* lncRNA in our designed ceRNA network. *Hsa-mir-497-5p* miRNA has been shown to inhibit tumor cell growth and invasion, and the expression of the *CASC9* lncRNA is abnormally elevated in multiple malignancies [27]. However, the precise mechanism of *ATP2A2* regulation in HCM progression needs further investigation.

## Conclusions

HCM is closely associated with the immune system. Hypoxia can modulate tissue dysfunction and disease development through immune cell dysregulation. Hypoxia is associated with myocardial fibrosis [28], which causes immune cell activation to affect cardiac function [11]. Thus, therapeutic regimens that target hypoxia and immune pathways may ameliorate HCM, improve cardiac function, and delay disease progression.

In the current study, we identified a ceRNA network with critical genes and three hypoxia- and immune-related genes (*ATP2A2*, *DDAH1*, and *OMA1*), which provides a theoretical foundation for the relationship between HCM and hypoxia and immunity.

### Supplementary Information

**Additional file 1: Table S1.** List of 493hypoxia genes.

**Additional file 2: Table S2.** Measuringresults in the hypertrophic cardiomyopathy mouse model.

**Additional file 3: Table S3.** Theregression coefficients of 11 gene in the least absolute shrinkage andselection operator (LASSO) COX regression model.

**Additional file 4: Table S4.** Lists for612 key module genes in five key modules selected using weighted geneco-expression network analysis (WGCNA).

**Additional file 5: Table S5.** Results forGene set enrichment analyses (GSEA) of three key genes.

**Additional file 6: Figure S1.** Verificationof three hypoxia- and immune-related genes in the combined datasets selectedfrom GSE141910 and GSE130036. (A) Before and (B) after batch correction. (C)The expression of three key genes in the combined cohorts (****P<0.0001, HCMvs. Control). (D) Receiver operating characteristic (ROC) analysis of three keygenes.

**Additional file 7: Table S6.** Relationshippairs between key genes and targeted miRNAs in miRWalk website.

**Additional file 8: Table S7.** Results for miRNA-lncRNAregulatory relationships through starbase website.

**Additional file 9: Table S8.** Transverse AorticConstriction (TAC) model in mice. Cardiac function indexes such as LVEF weredetected by echocardiography.

## Data Availability

The original contributions presented in the study are included in the article/Additional file, further inquiries can be directed to the corresponding authors.

## Abbreviations

AMI: Acute myocardial infarction
ATP2A2: ATPase sarcoplasmic/endoplasmic reticulum Ca2 + transporting 2
AUC: Area under curve
ceRNA: Competing endogenous RNA
DDAH1: Dimethylarginine dimethylaminohydrolase 1
DEGs: Differentially expressed genes
ECG: Electrocardiogram
FDR: False discovery rate
GEO: Gene expression omnibus
GO: Gene ontology
GSEA: Gene set enrichment analysis
HCM: Hypertrophic cardiomyopathy
HW/BW: Heart weight/body weight
HW/TL: Heart weight/tibia length
IVS: Interventricular septum
KEGG: Kyoto encyclopedia of genes and genomes
LVEF: Left ventricular ejection fractions
MCP: Microenvironmental cell population
ME: Module signature genes
MM: Module membership
MS: Module significance
OMA1: OMA1 zinc metallopeptidase
ROC: Receiver operating characteristic
ROS: Reactive oxygen species
ST: ST-segment
SVM-RFE: Support vector machine recursive feature elimination
TOM: Topological overlap matrix
WGCNA: Weighted gene co-expression network analysis
