# Supplementary figures and images for: Identification and analysis of key hypoxia- and immune-related genes in hypertrophic cardiomyopathy
Source: Biol Res. 2023 Aug 9;56:45. doi: 10.1186/s40659-023-00451-4 (PMC10410988; doi:10.1186/s40659-023-00451-4)

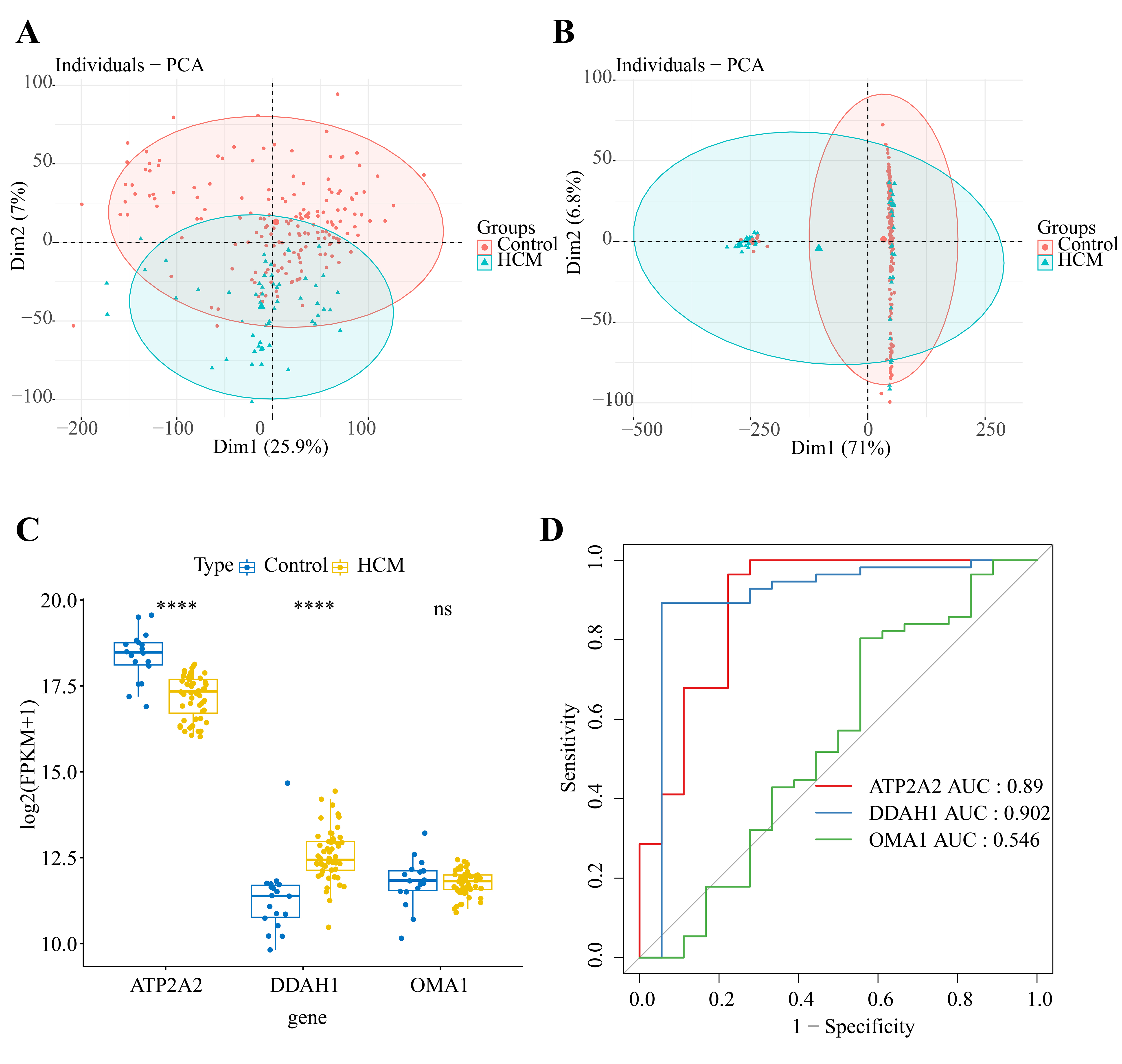

Supplement: Supplementary file 6 — Additional file 6: Figure S1. Verificationof three hypoxia- and immune-related genes in the combined datasets selectedfrom GSE141910 and GSE130036. (A) Before and (B) after batch correction. (C)The expression of three key genes in the combined cohorts (****P<0.0001, HCMvs. Control). (D) Receiver operating characteristic (ROC) analysis of three keygenes. [file 40659_2023_451_MOESM6_ESM.jpg]
